# The Public and Its Physic

**Published:** 1887-03-19

**Authors:** 


### THE PUBLIC AND ITS PHYSIC.

It is a portentous sign of the times that doctors are becoming more numerous and more necessary year by year. For the most part they are men of fair honesty and good brains; and it would be better for the world if they were employed at some productive labour, rather than at the mere tinkerings of small ills which now waste their energies and their time. It is pitiable, if not amusing, to see a stalwart fellow of six feet come, mountain like, out of a little pill-box of a brougham, and give a loud rat-a-tat-tat upon the knocker, as if he were a lady performing that most insipid and imbecile of all human duties—a morning call. Fine soldiers some of these same doctors would make, and others of them look as if they had been born for no other purpose but to swing a scythe in a cornfield, or to bring a refractory bullock to his senses with a blow of the fist. But the public has itself to thank. It will pay men for physic when it is ill: it will not pay them for guidance when it is well. The public is a fool for its pains. It is better to keep a house sound and weather-tight by painting and pointing than to let it fall into ruins and then to spend a fortune in repairs. Everybody says "a stitch in time saves nine," but nobody believes it when it is applied to physic.

Will the busy man or the woman in search of a time-killer, spare a minute for the consideration of a little easy philosophy? Think for a brief period of this fundamental proposition. What is the object of life; what is the use of it? The answer is twofold. We should get all the legitimate enjoyment out of the world we possibly can. That is one leg of our duty. We should give to the world the very best that is in us. That is the other. He who supports his mental and moral nature on these two limbs will have a very well-matched pair of legs. We will suppose these two propositions to be accepted. How do they affect the man who is sick when he might be well? They declare him to be a fool. Take a dyspeptic. Can he get any enjoyment out of life? A ton weight lies on his stomach after dinner; a colic twists a knot in his colon; a foul tongue poisons his breath in the morning, and a headache racks his brain. His temper is morose, his judgment perverted, his benevolence diminished, and his courage impaired. What pleasure can he get out of existence? None! To him the question, "Is life worth living?" has but one answer—"No."

If such a man can extract no pleasure out of life for himself, what can he give to others—to the world generally? He can give a good deal which the world would rather not have. To his wife—if, unhappily for her, he has one—he can be a marplot, a kill-joy, a wet blanket, a frying-pan to-day and a fire to-morrow; a whip and a scorpion by turns, a pest, a nuisance; a witch's cauldron in which every youthful hope, every domestic joy, every pleasure of life, is mingled with gall and bitterness, with misery and disgust. Or take the gouty man. When his enemy has him by the throat, or rather by the big toe, how does his barque fare on the swelling tide of life? His meats and his drinks, his wines and his sauces, rise up around him like so many evil spirits come to torment him before his time. "Life pleasant," he will say; "yes, if red-hot needles pushed into your joints be pleasure, then life is pleasant, no doubt."

Now these men need not be ill. As a rule, both the dyspeptic and the gouty man, as well as many other chronic ailers, have the matter in their own hands. If they would but find an intelligent and plainspoken doctor, and *pay him for teaching them how to keep well*, all would be right. But they will not. For—and here lies the root of the mischief—they would not *obey his teaching*, even if they paid him for the lessons. That is the secret of the whole business. The world pays a huge army of doctors to attend to it when it is sick, because it has not sense enough to learn and to practise the art of healthy living. Twelve hundred different drugs in the pharmacopœia! *Prodigious!* as Dominie Sampson would say. There need not be more than twelve, if everybody were moderately wise.

Is there any hope of better things? Undoubtedly. Children of both sexes must be taught how to live healthily. That is where the hope lies. They need not be taught, as they now are, a useless smattering of physiology and chemistry. That is the merest waste of time. The world has now gathered and formulated a body of sound "principles of health," the results of the experience of all men, in all countries and all ages. Let these principles be brought together, clearly stated, and simply illustrated; and let them be made one branch of study in every school throughout the kingdom—from the highest to the lowest. Let every teacher's college have its accomplished professor of the science of healthy life; and let the instructors of youth receive a thorough training in that as in other subjects. What would be the result? A revolution in the habits of our people. The production of a robuster race. A new epoch in the history of the nation. Fresh life, fresh power, fresh hope for the Empire and the world.

